# Dissecting *R* gene and host genetic background effect on the *Brassica napus* defense response to *Leptosphaeria maculans*

**DOI:** 10.1038/s41598-019-43419-9

**Published:** 2019-05-06

**Authors:** Parham Haddadi, Nicholas J. Larkan, M. Hossein Borhan

**Affiliations:** 1 Agriculture and Agri-Food Canada, Saskatoon Research and Development Centre, 107 Science Place, Saskatoon, SK S7N 0X2 Canada; 2 Armatus Genetics Inc., Saskatoon, SK S7J 4M2 Canada

**Keywords:** Molecular biology, Molecular biology, Plant stress responses, Plant stress responses

## Abstract

While our understanding of the genetics underlying the *Brassica*-*Leptosphaeria* pathosystem has advanced greatly in the last decade, differences in molecular responses due to interaction between resistance genes and host genetic background has not been studied. We applied RNAseq technology to monitor the transcriptome profiles of *Brassica napus* (*Bn*) lines carrying one of four blackleg *R* genes (*Rlm2*, *Rlm3*, *LepR1* & *LepR2*) in Topas or Westar background, during the early stages of infection by a *Leptosphaeria maculans* (*Lm*) isolate carrying the corresponding *Avr* genes. We observed upregulation of host genes involved in hormone signalling, cell wall thickening, response to chitin and glucosinolate production in all *R* gene lines at 3 day after inoculation (dai) albeit having higher level of expression in *LepR1* and *Rlm2* than in *Rlm3* and *LepR2* lines. *Bn-SOBIR1* (Suppressor Of BIR1-1), a receptor like kinase (RLK) that forms complex receptor like proteins (RLPs) was highly expressed in *LepR1* and *Rlm2* at 3 dai. In contrast *Bn-SOBIR1* induction was low in *Rlm3* line, which could indicate that *Rlm3* may function independent of *SOBIR1*. Expression of Salicylic acid (SA) related defense was enhanced in *LepR1* and *Rlm2* at 3 dai. In contrast to SA, expression of *Bn* genes with homology to *PDF1.2*, a jasmonic acid (JA) pathway marker, were increased in all *Rlm* and *LepR* lines at 6 and 9 dai. Effect of host genetic background on induction of defense, was determined by comparison of *LepR1* and *LepR2* in Topas vs Westar genotype (i.e. T-*LepR1* vs W-*LepR1* and T-*LepR2* vs W-*LepR2*). In both cases (regardless of *R* gene) overall number of defense related genes at the earliest time point (3 dai) was higher in Tops compared to Westar. SA and JA markers genes such as *PR1* and *PDF1.2* were more induced in Topas compared to Westar introgression lines at this time point. Even in the absence of any *R* gene, effect of Topas genotype in enhanced defense, was also evident by the induction of *PDF1.2* that started at a low level at 3 dai and peaked at 6 and 9 dai, while no induction in Westar genotype was observed at any of these time points. Overall, variation in time and intensity of expression of genes related to defense, was clearly dependent on both *R* gene and the host genotype.

## Introduction

Plants are exposed to a myriad of microorganism in their environment. However, natural physical barriers and chemical deterrents guard the plants from the majority of these microbes. The small number of microorganisms that overcome this passive defense still have to evade recognition by the plant cell surface and cytoplasmic receptors that have evolved to perceive conserved structural molecules and virulence factors termed pathogen associated molecular patterns (PAMP) and effectors, respectively^1–3^. Effector-triggered immunity (ETI) is often manifested as a rapid and strong defense response leading to induction of cell death, also known as hypersensitive response (HR), at the site of infection to arrest pathogen growth beyond the point of entry. HR provides early and robust resistance against *Leptosphaeria maculans* (*Lm*), the hemibotrophic fungal agent of blackleg disease in canola (oilseed rape, *Brassica napus*; *Bn*). The gene expression profile of *Bn* cotyledons infected with *Lm* reveals the transition from biotrophy to necrotrophy as infection progresses in a compatible interaction^4^. Genes related to salicylic acid (SA) pathway are induced at the earlier stages of infection (3 days after inoculation; dai) while expression of jasmonic acid (JA) pathway genes, linked to plant response to necrotrophic pathogens, is up-regulated at the later stages (6–9 dai). Induction of JA defense correlates with the up-regulation of the *Lm* gene necrosis and ethylene‐inducing peptide‐1 (*Nep‐1*) ‐like protein, a well-known marker of transition from biotrophy to necrotrophy in fungi^4^. Becker *et al*.^5^ also reported the importance of SA and JA pathways in a *Bn* line carrying the *LepR1* resistance gene in response to a *Lm* isolate carrying the corresponding *AvrLep1* effector. Upon perception of *Lm* an array of host genes functionally-defined as proteases and protease inhibitors, chitinases, peroxidases, transcription factors (WRKY, AP2/EREBP, MYB), genes related to the production of the secondary metabolites and genes involved in plant cell wall reinforcement were differentially expressed^4,5^.

Despite being a qualitative trait, the immunity response triggered by *R* gene *Avr* gene recognition varies in phenotype; from a highly-localised response seen as minute necrosis, trailing necrosis, no visual symptoms or contained pathogen growth and sporulation^6^. Both genotype of the host and functional variation of *R* genes cause variation in the interaction phenotype^7^. R protein activation and recognition of pathogen effector proteins often requires inter and/or intra-molecular interaction and complex formation with other host proteins^8,9^. These receptor complexes affect R protein function and consequently plant pathogen interaction phenotype^8,9^. Natural variation in Arabidopsis has served as a tool to dissect the genetic basis of polymorphism in plant to interaction with pathogens^10,11^.

Race specific *R* genes have been widely used in breeding for *Brassica napus* (canola; oilseed rape) resistance to blackleg disease caused by the ascomycete fungus *Leptosphaeria maculans*^12^. Out of nineteen *R* genes reported from Brassica species, eleven of them (*Rlm1*, *Rlm2*, *Rlm3*, *Rlm4*, *Rlm7*, *Rlm9, Rlm*11, *LepR1*, *LepR2*, *LepR3* and *LepR4*) originated from the A genome of *Bn* and *Brassica rapa* (*Br*)^12,13^. The genetics of the *Bn-Ln* pathosystem has been greatly advanced by the cloning of *Lm Avr* genes; *AvrLm1, 2, 3, 4*–*7, 5*–*9, 6*, and *11*^14–22^ and the characterisation of the *Bn R* genes *LepR3* and *Rlm2*^23,24^. Host differential lines are indispensable for genotyping plant pathogen races. In previous studies, *Rlm* and *LepR* genes were introgressed into common susceptible *Bn* doubled-haploid lines Topas (DH16516) or Westar (N-o-1)^13,25^. This led to generation of seven Topas introgression lines (T-*Rlm1*, T-*Rlm2*, T-*Rlm3*, T-*Rlm4*, T-*LepR1*, T-*LepR2*, T-*LepR3*), which share 92.9–98.9% of their genomic background with the susceptible parental lines^13^, and two Westar introgression lines (1065; W-*LepR1*, 1135; W-*LepR2*). These introgression lines provide a unique tool to compare the function of different *R* genes in a common genotype background and also for the dissection of the effect of host genetic background on the defense responses triggered by the same *R* gene. To gain insight into the molecular mechanism of host genetic background and *R* gene effect we conducted a comprehensive transcriptome analysis by performing RNAseq (1.5 billion raw reads- 168 samples) on *Lm* infected cotyledons at 0, 3, 6 and 9 dai. Here we describe the differences in gene expression profiles and defense pathways among these treatments and their correlation with the variation in visual interaction phenotypes.

## Results

### Variation of *R* gene phenotypic response to *Lm* infection

We have introduced individual *Rlm* (resistance to *L. maculans*) and *LepR* (*Leptosphaeria resistance*) genes into the common susceptible *Bn* cultivar Topas using repeated backcrossing and selfing (BC5S3) that allowed generation of individual Topas lines each harbouring *Rlm* or *LepR* gene intervals which was confirmed by genome wide high density SNP profiling of each line, minimizing background effects on *R* gene performance^13^. *Bn* cultivars Topas and Westar being completely susceptible to *Lm* are routinely used as control in blackleg pathology tests in the greenhouse and field trials. We have previously noted that the cotyledon immune response triggered by *R* genes in Topas is often more robust compared to the immune response induced by the same *R* gene in Westar^13^. To explore the genetics of this variation, we studied the effect of *R* genes and host genetic background on the global gene expression in response to *Lm* infection.

**Figure 1:**
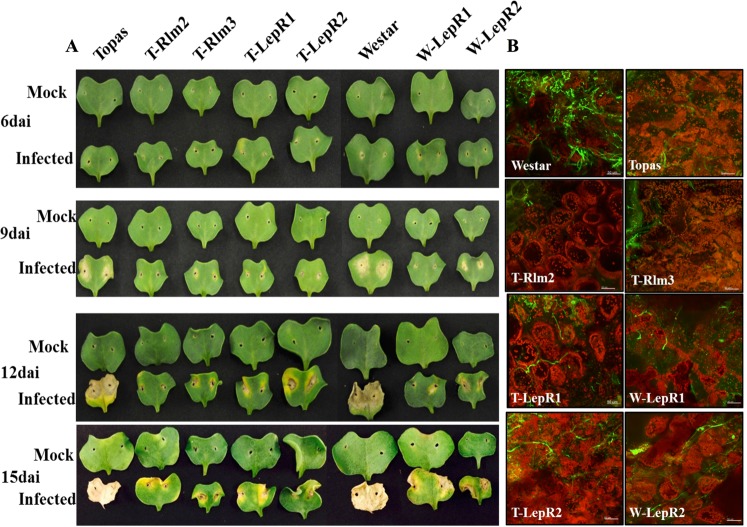
Disease symptoms on inoculated cotyledons of *B. napus cv*. Topas, Westar and Introgression Lines (ILs) with *Leptosphaeria maculans* (*Lm*). (**A**) Cotyledons of Topas, Westar and ILs photographed at 6, 9, 12 and 15 days after inoculation (dai). (**B**) *Lm* hyphae (green) shown by alexa fluor 488 (AF488) conjugate of wheat germ agglutinin (WGA) through field of view (FOV) at lesion on infected cotyledons of Topas, Westar and ILs at 6dai.

Parallel comparisons were made between Topas introgression resistance lines (T-*Rlm2*, T-*Rlm3*, T-*LepR1* and T-*LepR2*) with *Bn* cv 1065 and 1135 harbouring *LepR1* and *LepR2* genes, respectively, in Westar background (W-*LepR1* and W-*LepR2*). This allowed to study the effect of plant genotype on the dynamics of host defense against *Lm*. Plants were inoculated at the seedling stage with the pycnidiospores of *Lm* isolate 00–100 (Avr phenoytype A2-A3-A5-A6-(8)-A9-(10)-AS-AL1-AL2-(L4))^13^ and RNAseq was conducted on samples collected at 3, 6 and 9 dai (Supp. Table 1). Concomitantly visual and microscopy phenotypic responses were observed at 3, 6, 9, 12, 15 dai. There were no visible symptoms at 3 dai. At 6 dai a chlorotic ring surrounding the inoculation site became clearly visible which later expanded, leading to the formation of lesion and tissue collapse around the site of inoculation in Topas and Westar while in the Topas introgression lines (T-*Rlm2*, T-*Rlm3*, T-*LepR1* and T-*LepR2*) pathogen growth was contained within the inoculation site (Fig. 1). The hypersensitive response (HR) appeared less intense at 6 dai in the Westar introgression lines (W-*LepR1* and W-*LepR2*) compared to T-*LepR1* and T-*LepR2*, judging by the intensity of brown tissue surrounding the inoculation site.

### RNA sequencing and gene expression profile during infection

**Figure 2:**
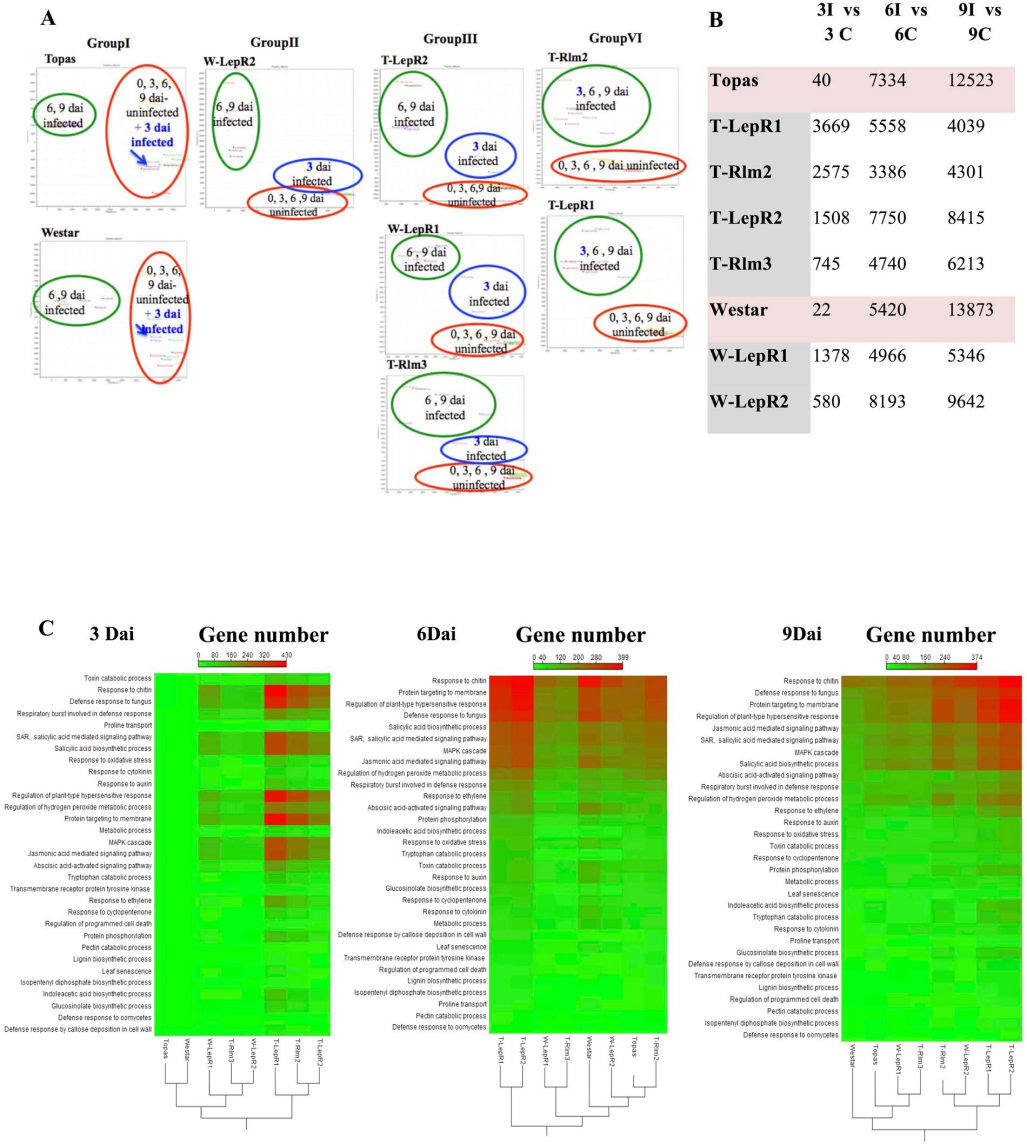
Global evaluation of RNA-seq and defense response to *Leptosphaeria maculans* (*Lm*) in Introgression Lines (ILs) compared to Topas and Westar at 3, 6 and 9dai. (**A**) PCA displays clear distinction between the transcriptome of resistance lines and wild parents at different time points. (**B**) Differentially expressed genes (DEGs) obtained at various time points in Topas, Westar and ILs (**C**) GO term enrichment. Intensity scale: Red represents more up-regulated genes corresponding to GO term in response to *Lm*. ‘3I/3C’ means ‘3 dai for inoculated with *Lm*/3 dai for not inoculated control’. ‘6I/6C’ means ‘6 dai for inoculated with *Lm*/6 dai for not inoculated control’. ‘9I/9C’ means ‘9 dai for inoculated with *Lm*/9 dai for not inoculated control’.

RNA reads from three biological replicates (1.5 billion paired-end reads from infected and mock-inoculated cotyledons) were analysed according to the methods described previously^4^. The number of reads for each sample is presented in the Supp. Table 1. Variability among the samples was determined by conducting principal component analysis (PCA). PCA displayed clear distinction between the transcriptome of introgression lines (ILs) and the susceptible parental lines at different time points (Fig. 2A). PCA distinguished four separate groups. “Group I” consisted of Topas and Westar in which transcript profiles of *Lm* infected lines at 3 dai grouped under the same cluster as transcript profiles of mock-inoculated Topas and Westar 3, 6 and 9 dai. Transcript profiles of *Lm* infected T-*LepR2*, T-*Rlm3* and W-*LepR1* at 3 dai formed a cluster separated from the transcript profiles of mock-inoculated lines (3, 6 and 9 dai) and *Lm* infected lines at 6 and 9 dai (Group III). Transcript profile of *Lm* infected W-*LepR2* at 3 dai overlapped with the cluster containing transcript profiles of the mock-inoculated lines at 3, 6 and 9 dai (Group II). Comparison of all the differentially expressed genes (DEG) among the various lines and time points also revealed a delay in response for the two *Bn* susceptible cultivars (Topas and Westar). Group VI, composed of T-*LepR1* and T-*Rlm2* indicated a more rapid defense response by *LepR1* and *Rlm2* evident by clear distinction between transcript profiles of *Lm* infected lines at (3, 6 and 9 dai) and transcript profiles of mock-inoculated lines at the same time points (Fig. 2A). At 3 dai, fewer genes were differentially expressed in Topas and Westar than in ILs (40 and 22, respectively) while the total number of DEG in the ILs were between 580 and 3669 for the same time point (Fig. 2B).

The most notable and rapid changes in gene expression profile of ILs (3 dai) were changes in expression of genes with known function in plant defense such as response to chitin, oxidative burst, and salicylic acid (SA), jasmonic acid (JA), abscisic acid (ABA), ethylene and glucosinolate pathways. In comparison, for the susceptible Westar and Topas lines, changes in expression of defense pathway-related genes occurred at 6 dai (Fig. 2C). This latent differential gene expression response to infection is depicted in the PCA analysis (Fig. 2A).

### Key defense response triggered by *R* genes

To define common defense pathways triggered by the race-specific resistance genes in *Bn* in response to *Lm* we compared the early defense response (3 dai) profile of gene expression across all the ILs. This comparison further validated the key genes involved in seedling resistance to *Lm*^4^. The pattern of DEG in all ILs indicated that early detection of *Lm* by *Bn* occurs through the activation of cell surface receptors. Examples of these were Rlm2/LepR3 and their paralogues, several Wall-Associated Kinases (WAKs) and Cysteine-rich Receptor-like Kinases (CRKs) containing DUF26 (domain of unknown function 26) as well as BIR1 and SOBIR1 that are components of PAMP/Effector receptor complexes^25,26^. In this study our data showed strong expression of six copies of *Bn SOBIR1* homologues in T-*LepR1* and T-*Rlm2*, peaking at 3 dai, which was also observed in T-*LepR2*, W-*LepR1* and W-*LepR2* but to lesser extent, compared to the susceptible controls, Westar and Topas, in which *SOBIR1* expression was not induced at 3 dai (Supp. Fig. 1A). Expression of only two out of the six *SOBIR1* homologues was induced in T-*Rlm3* albeit to a much lower level compared to the other *R* gene introgression lines (Supp. Fig. 1A). Our data also showed the involvement of some of the CRK family of cell surface receptors in response to *Lm* at the earlier time points of infection. Among the differentially expressed CRKs with a role in plant defense were homologues of the Arabidopsis genes *CRK 4*, *5* and *20*, which act as HR inducers^27,28^ (Supp. Fig. 1B) and *CRK 2*, *10* and *11*, involved in chitin-triggered defense response^29^ (Supp. Fig. 1C).

**Figure 3:**
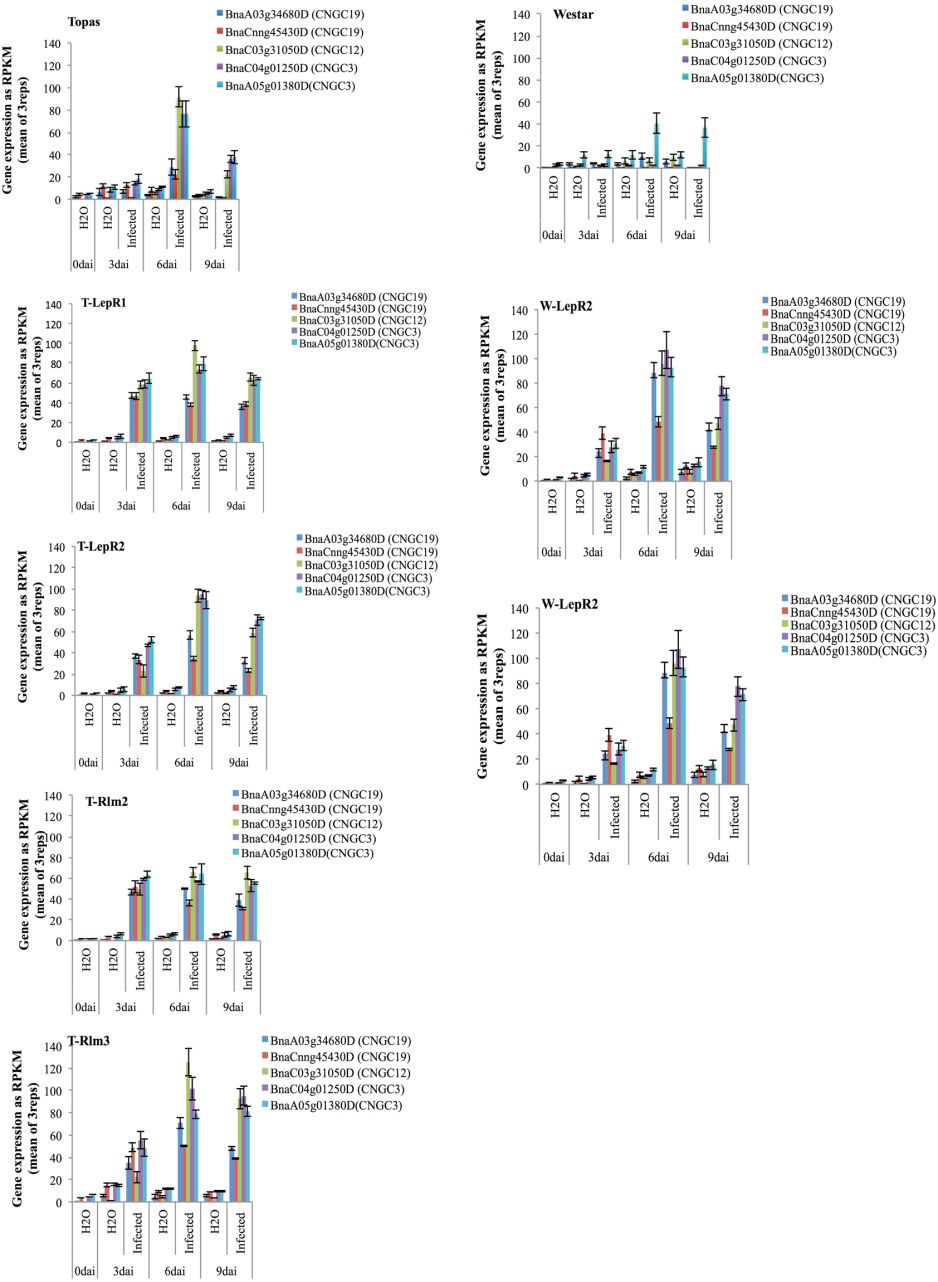
Expression profile of genes encoding for Cyclic Nucleotide-Gated Ion Channels (CNGC) that are likely involved in influx of calcium in Topas, Westar and Introgression Lines (ILs) at 3, 6 and 9dai.

Perceived signals of *Lm* are relayed through the activation of genes encoding for Cyclic Nucleotide-Gated Ion Channels (*CNGC*) that are likely involved in influx of calcium. *CNGC 3*, *12* and 19 have been reported to be involved in plant immunity^30^. Transcripts of *CNGC 3*, *12* and *19* homologues were enriched in response to *Lm* at 3 dai in the ILs while these genes were not induced in Topas until 6 dpi (Fig. 3). No induction of *CNGC* genes was observed for Westar with the exception of *CNGC*3 (BnaA05g01380D) (Fig. 3). Three genes (BnaA04g09500D, BnaC03g25880D and BnaC06g12520D) annotated as Calcium-binding EF-hand family proteins were also up-regulated at 3 dai.

Comparison of defense pathway genes common among the *R* genes shows that SA is the main plant hormone involved in the response and is activated in earlier time points (3 dai) in all of the ILs. In total 72 genes showed a strong positive correlation (>0.8) with genes reported to be induced in response to SA (Supp. Fig. 2). The most prominent SA dependent genes were *EDS1*, *FMO1*, *EDS5*, *NIM1*/*NPR1* interacting protein 1 and 2 (*NIMIN1* and *NIMIN2*, Supp. Fig. 3A), *PAD4*, *PR1*, *PR2*, as well as several WRKY transcription factors, in particular *WRKY18* and 70 (Supp. Fig. 3B) that are reported to be positive regulators of the SA pathway^31^. This rapid SA dependent defense response was followed by the induction of antifungal enzymes such as chitinases (CHI) (Supp. Fig. 3C). At later time points (6 and 9 dai), DEG related to JA and auxin (Aux) pathways showed positive and strong correlation although SA pathway was still active at these time points, but to a lesser extent (Supp. Fig. 2).

In addition to Flavin-containing Monooxygenase (*FMO*), up-regulation of genes such as *ALD1* (BnaA03g38440D and BnaC0345280D, infected vs mock; RPKM = 120 vs 0 at 3 dai) and amino acid transporter Lys/His transporter 1 and 7 (BnC01g03360D and BnaAnn11580D, infected vs mock; RPKM = 113 vs 0 at 3 dai) indicated the early potentiation and systemic spread of defense signals, prompting the systemic acquired resistance (SAR) response^32–34^. Arabidopsis *ALD1* (*agd2*-like Defense Response Protein 1) mutants are reported to be impaired in SAR, susceptible to bacteria pathogens and compromised in defense against *P. syringae*^35^. Two *BAP2* (BON1 Associated Proteins) genes (BnaA03g56970D and BnaC03g25440D) were up-regulated at 3 dai (70 RPKM in ILs compared to 0 to 0.2 RPKM in all mock control at 3, 6 and 9 dai). The expression level of *BAP2* in infected tissue decreased at 6 and 9 dai to 10–20 RPKM. BAP2 is a negative regulator of cell death and its over-expression, along with BON1, prevents cell death^36^.

**Figure 4:**
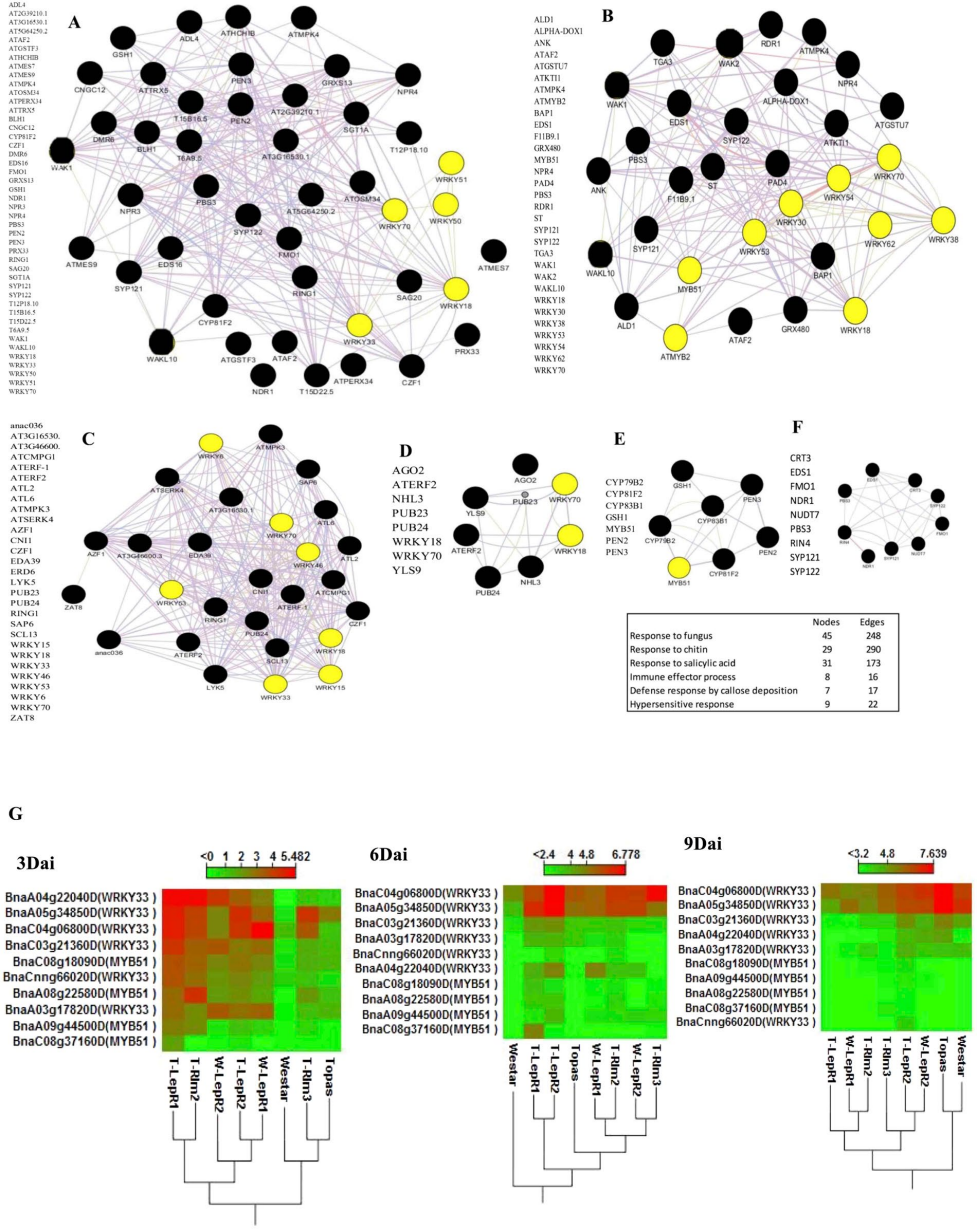
Predicted networks these are involved in early response (3 dai) to *Leptosphaeria maculans* (*Lm*) in Introgression Lines (ILs). A predicted network that is involved in (**A**) response to fungus, (**B**) response to salicylic acid, (**C**) chitin response, (**D**) immune effector process, (**E**) defense response by cell wall thickening or callose deposition, (**F**) hypersensitive response at 3dai. TFs are highlighted in yellow. (**G**) Heatmaps present the difference in expression of *B. napus* genes with homology to WRKY33 and MYB51 in *Lm*-inoculated compared with mock-treated cotyledons of Topas, Westar and ILs at 3.6 and 9 dai. The colors correspond to log 2 RPKM (Infected – Uninfected) ranging from red (high) to green (low). Euclidean distance for the distances measure and complete linkage for clusters linkage criteria were selected.

We predicted gene networks associated with response to fungi, response to chitin, response to salicylic acid, defense response by callose deposition and transcriptional regulatory elements involved in response to *Lm* based on co-expressed genes (Fig. 4A–F). Among the differentially expressed transcription factors with a role in plant defense were *WRKY*, *AP2/EREBP* and *MYB*. Expression of several WRKY transcription factors were up-regulated in the ILs. Transcripts of *WRKY 70, 51, 50, 33, 18* associated with response to fungi and *MYB51, MYB2* and *WRKY 62, 53* related to salicylic acid response were enriched by 3 dai in the ILs. Among several *WRKY* genes, *WRKY33* and *MYB51* (predicted transcriptional regulator for glucosinolates) showed the highest expression difference to mock in the ILs (Fig. 4G).

### Genetic background and *R* gene effect on the host defense response

**Figure 5:**
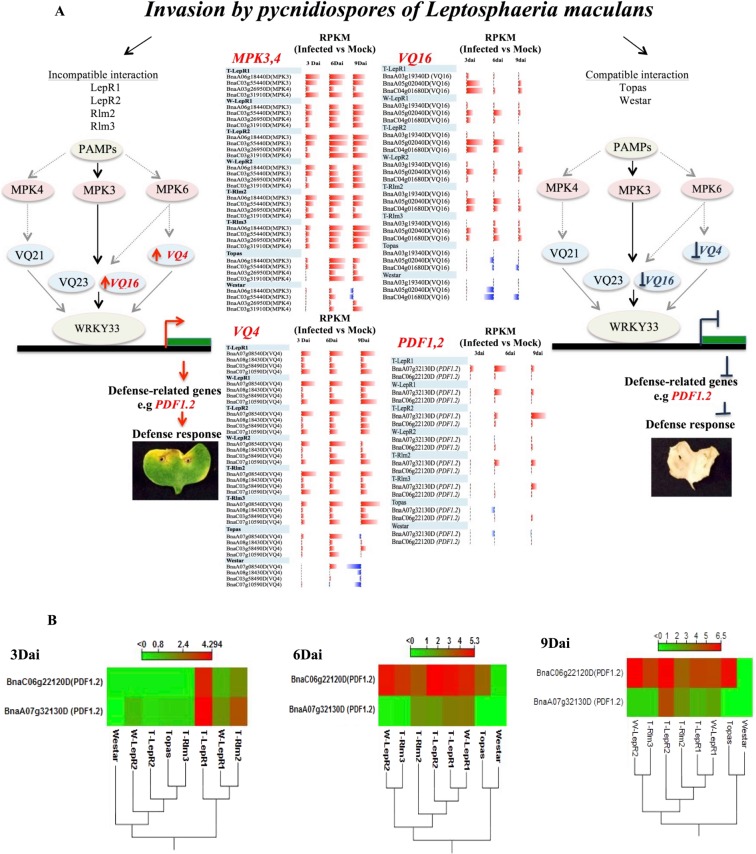
(**A**) An overview of MPK-VQ-JA signaling in *Brassica napus* and *Leptosphaeria maculans* (*Lm*) interaction. Identification of networks involved in earlier response to blackleg in ILs provided the evidence that WRKY33 is involved in response to *Lm*. Accumulation of transcripts associated with *WRKY33*, *VQ16* and *VQ23* homologues were enriched by 3 dai in resistance lines. RNAseq analysis revealed all 3 copies of *VQ16* is suppressed after invasion by pycnidiospores of *Leptosphaeria maculans* in Topas and Westar, consequently one copy of *PDF1.2* is highly suppressed in susceptible lines whilst it is highly accumulated in resistance lines. (**B**) Heatmaps present the difference in expression of *B. napus* genes with homology to JA (*PDF1.2*) in *Lm*-inoculated compared with mock-treated cotyledons of Topas, Westar and resistance lines at 3, 6, 9 dai. The colors correspond to log 2 RPKM (Infected – Uninfected) ranging from red (high) to green (low). Euclidean distance for the distances measure and complete linkage for clusters linkage criteria were selected.

Clustering of ILs and their respective susceptible lines, based on the number of differentially expressed genes, provided an overview and comparison of the dynamics of defense in each of these lines. Little change in gene expression was detected in the two susceptible lines Topas and Westar at the earliest time point (3 dai, Fig. 2B). The most enhanced gene expression (in terms of the total number of DEG) occurred in T-*LepR1* followed by T-*Rlm2*, T-*LepR2* and W-*LepR1* (Fig. 2B). In W-*LepR2* and T-*Rlm3*, the total number of DEG was significantly reduced showing a transcriptome profiles between the susceptible and T*-LepR1*, T*-Rlm2*. Genes related to proteins targeted to the membrane, defense against fungi, response to chitin, Reactive Oxygen Species (ROS), Systemic Acquired Resistance (SAR), SA, JA and genes involved in hypersensitive response, glucosinolate pathway and callose deposition were most prevalent in ILs (particularly in T-*LepR1* and T-*Rlm2*) at 3 dai (Fig. 2C). In comparison, in Topas and Westar enrichment for genes related to these pathways occurred at 6 dai (Fig. 2C). All of these genes continued to be differentially expressed in T-*LepR1*, T-*LepR2* and T-*Rlm2* until 9 dai (Fig. 2C). At 3 dai, overexpression of marker genes related to SA (*PR1*, *WRK70*, *ICS1*) and JA (*PDF1*) were noticed in all ILs, however it was again most enhanced in T-*LepR*1 and T-*Rlm2* (Supp. Fig. 3B and Fig. 5). Up-regulation of these marker genes occurred at 6 and 9 dai in all other *R* lines as well as Topas (Supp. Fig. 3B and Fig. 5). Induction of SA marker genes in Westar started at 6 and 9 dai however with much lower expression level as compared to Topas (Supp. Fig. 3B). Interestingly *PDF1*, the marker for JA pathway, was not upregulated in Westar at any of these time points (Fig. 5).

A search of the KEGG database using DEG from T*-LepR1*, T*-LepR2*, T*-Rlm2* and T*-Rlm3* at 3 dai was performed. Pathways related to metabolic processes involved in energy production, such as glycolysis and the pentose phosphate pathway, TCA cycle, mitochondrial electron transport, ATP biosynthesis, and biosynthesis of some amino acids such as lysine and methionine, whose catabolism leads to energy production^37^, as well as biosynthesis of glutamic acid, arginine, serine, and glycine, which are associated with photorespiration^38^ were highly activated (based on the number of DEG associated with these pathways) in T-*LepR1* and T-*Rlm2* as compared to T-*LepR2* and T-*Rlm3*.

Pathogens rely on host-derived nutrients for their growth. Balance of influx and efflux of nutrients between the source and sink tissue either promotes or limits pathogen growth^39^. We searched for the DEGs encoding nutrient transporters and noticed that nitrate, sucrose and amino acid transporters were the most abundant among the DEG in T-*Rlm2* and T-*LepR1* followed by T-*LepR2* and T-*Rlm3*. In addition, there was a noticeable reduction in the number of nutrient transporters in W-*LepR1* and W-*LepR2* compared to T-*LepR*1 and T-*LepR2*, respectively. A search of plant defense-related GO terms revealed saturation for T-*Rlm2* and T-*LepR1* followed by T-*LepR2* and T-*Rlm3* and a significant reduction in W-*LepR1* and W-*LepR2*. The most notable defense-related GO terms were related to cell wall modification (e.g. callose deposition, pectin metabolism), HR response, oxidative burst in response to defense, SA and glucosinolate biosynthesis, SA, JA, ABA, and SAR mediated signaling pathways, defense response to pathogens, response to chitin and response to toxins (Fig. 2C). Judging by the phenotypic response (Fig. 1A) *Rlm2* and *LepR1* display a more robust and rapid resistance response than *Rlm3* and *LepR2*. Similarly as described above, pattern of DEG indicated a rapid response in defense and coordinated induction of pathways that provide energy and nutrient during the induction of defense in T- *Rlm2* and T- *LepR1* compared to T-*LepR2* and T-*Rlm3*. This pattern of gene regulation occurred at 6 dai in *Rlm3* and *LepR1* lines. Likewise, effect of host genetic background on defense response initiated by the same *R* gene was evident from the prevalence of DEG related to biotic stress in T-*LepR1* and T-*LepR2* compared to W-*LepR1* and W-*LepR2*, respectively.

### Comparison of defense response in Topas and Westar with and without *R* genes

We have previously observed that some *R* genes in the Topas background show an enhanced resistance response compared to the level of resistance when the same *R* gene is expressed in Westar^13^. Also, development of lesions in Topas at the earlier stages of infection is slower compared to Westar, although at the late phases of infection (10–14 dai) development of lesion in both cultivars is comparable. HORMONOMETER software^40^ was used to catalogue hormone defense networks in response to *Lm* infection in which the DEGs as the input query genes were compared against a database of Arabidopsis genes expressed in response to the application of different hormones. Comparison between the gene expression profile of Westar and Topas at 3 dai revealed a positive correlation between DEG related to SA and JA pathways in Topas at 3 dai while expression of genes related to these pathways showed a negative correlation for the same time point in Westar, indicating the importance of SA and JA in boosting the defence response in Topas (Supp. Fig. 4). A network of differentially expressed transcription factors generated by SeqEnrich^41^ showed the enrichment for phytoalexin camalexin in Topas vs Westar at 3 dai (Supp. Fig. 5A,B). Among the genes with confirmed role in defense, expressed in Topas but absent from Westar at 3 dai, were genes with homology to the Arabidopsis Extensin (AT1G21310), a hydroxyproline-rich glycoprotein (HRGPs), with reported roles in plant defense through strengthening of the plant cell wall^42,43^. High expression of several chitinase genes at 3 dai was detected in Topas (Supp. Fig. 3C). Another DEG in Topas with antimicrobial activity was a GDSL LIPASE reported to function in disrupting fungal spores as well as inducing SAR^44^. Induction of defense pathways in Topas and Westar were further examined when the same *R* gene was present in both backgrounds. Comparison of DEG in T-*LepR1* and W-*LepR1* and similarly between T-*LepR2* and W-*LepR2* provided further insight into molecular mechanisms of enhanced resistance in Topas vs Westar. Although presence of *LepR1* and *LepR2* in Westar triggered the induction of the main defense hormones, notably SA and JA as well as auxin, Et and brassinosteroid pathways, the overall number of DEG related to these pathways were higher in T-*LepR1* and T-*LepR2* compared to W-*LepR1* and T-*LepR2*, respectively (Supp. Fig. 4). A search of the KEGG database with DEG in T-*LepR1* and W-*LepR1* revealed that a serine/threonine kinase (OX1) is expressed at 3 dai in T-*LepR1* but not in W-*LepR1*. Ox1 is required for basal resistance and activation of MPK3 and MPK4^45^. Genes related to the glucosinolate pathway were induced in T-*LepR1* at 3 dai but not in W-*LepR1*. The brassinazole resistance (*BZR1*) gene (BnaA06g13460D) was suppressed in T-*LepR1*. It has been shown that silencing *BZR1* improved tobacco plants’ resistance to tobacco mosaic virus^46^. Activation of *BZR1* resulted in impaired PAMP-triggered ROS production and enhanced susceptibility to adapted and non-adapted strains of the bacterium *Pseudomonas syringae*^47^.

## Discussion

Plant response to pathogen infection leads to significant changes in the plant’s transcript profile. Despite overlaps and commonality between responses of different plant species to various pathogens, variation in plant phenotypic and molecular interactions to pathogens also occurs due to differences in *R* genes and host genetic backgrounds. Natural variation in Arabidopsis has been exploited to capture genetics for defense polymorphism against many oomycete, fungal and bacterial pathogens^48^. For some pathogens such as *Lm*, genetics and genomics of the defense response needs to be investigated using its natural host, as Arabidopsis does not provide an ideal model system^49^. Here we have taken advantage of several well-defined introgression lines each harboring individual *R* genes against blackleg pathogen in the *Bn* cv Topas, a common susceptible genotype. We monitored phenotypic interaction and changes in gene expression profile due to immunity response triggered by the race-specific resistance genes *Rlm2*, *Rlm3*, *LepR1* and *LepR2* in two susceptible *Bn* genetic background, Topas and Westar.

Comparing the transcript profiles of Topas and Westar and also W-*LepR1* with T-*LepR1* and W-*LepR2* with T-*LepR2* revealed a delay in defense response in Westar compared to Topas. While at the earliest time point i.e. 3 dai, changes in a limited number of defense-related genes were noticed in Topas, the transcript profile of infected Westar plants was the same as Westar mock inoculated controls. Similarly, judging by the defense related GO terms, immunity triggered by *LepR1* and *LepR2* was less intense in the Westar background compared to the immunity response induced by these same genes in Topas. Early induction of genes related to cell wall strengthening and production of antifungal compounds such as chitinases and lipases occurred in Topas but was not observed in Westar. The GO enrichment of camalexin in Topas early in infection was observed as compared to Westar (Supp. Fig. 5A,B). An Arabidopsis Phytoalexin Deficient 3 (*pad3*) mutant^50^ that is defective in camalexin production has been reported to be more susceptible to *Lm* infection^51^. The combined effect of *R* gene and host genetic background in generating a stronger defense response was also evident by expansion of DEG for SA, JA and Et pathways in T-*LepR1*, T-*LepR2* compared to W-*LepR1* and W-*LepR2*, respectively. Clustering of *R* gene ILs confirmed the boosting effect of the Topas genetic background on the level of gene expression at 3 dai for *LepR1* in Topas compared to *LepR1* in Westar. Differentially expressed genes in Topas vs Westar background could prove to be useful as markers to select the most suitable genotype as a recipient parent when developing *Bn* cultivars with resistance to *Lm*. The effect of *R* genes on spontaneity and robustness of defense response was measured based on the number and scope of defense related genes by comparing differentially expressed genes in T-*Rlm2*, T-*Rlm3*, T-*LepR1* and T-*LepR2*. Based on the microscopy observation of the strength of the interaction phenotype and the genes’ ability to limit the pathogen growth over the course of infection, these *R* genes could be ordered as T-*LepR1*/T-*Rlm2*; T-*LepR2* and finally T-*Rlm3* from the most robust to a weaker defense response. As shown in the heat map of DEG at 3 dai (Supp. Fig. 3C), in T-*LepR1*, T-*Rlm2* and to certain extent in T-*LepR2*, a strong induction of chitin-responsive genes, a known PAMP, and induction of genes related to callose deposition, up-regulation of ROS related genes, induction of SA and JA and to a lesser extent glucosinolate pathways, regulation of hypersensitive response and induction of downstream MAP kinases were the most prominent and well-documented indicators of plant immunity responses. It was only at 6 dai that these pathways were up-regulated to the same extent in T-*LepR2* however, in the case of T-*Rlm3*, expression of defense related pathways was significantly less than in the other Topas introgression lines.

PAMP triggered immunity (PTI) provides basal defense upon detection of conserved pathogen molecules while effector triggered immunity (ETI) provides a rapid and strong defence in response to pathogen virulence (effector) genes. We investigated the PTI and ETI defense against *Lm* by comparing the DEG with the Arabidopsis genes associated with PTI and ETI as reported by Dong *et al*.^52^. This comparison revealed strong induction of PTI and ETI related genes in T-*LepR1* and T-*Rlm2* (Supp. Fig. 6A). It has also been reported that Non-race Specific Disease Resistance 1 (NDR1) is a conserved downstream regulator of *R* signalling^53^. Interaction of NDR1 and RIN4 results in transduction of extracellular pathogen-derived signals^53^. The difference in expression of *Bn* genes with homology to *NDR1* related to ETI^54^ in *Lm*-inoculated compared with mock-treated cotyledons of Topas, Westar and the ILs revealed activation of these genes in incompatible hosts at 3 dai, with comparatively higher activity in T-*Rlm2*, T-*LepR1* and T-*LepR2* (Supp. Fig. 6B).

Among several WRKY transcription factors, WRKY11 and WRKY17 have both been reported to be involved in JA–dependent defense response^55^. Our result showed up-regulation of both in the ILs as compared to Topas and Westar. The WRKY33 transcription factor is reported to be important for plant resistance to hemibiotrophic and necrotrophic pathogens and to be involved in response to chitin, production of secondary metabolic and the phytoalexin biosynthetic pathway (Fig. 4A,C). Bimolecular fluorescence complementation previously revealed that WRKY33 interacts with nuclear-encoded SIGMA FACTOR BINDING PROTEIN 1 (SIB1) and SIB2^56^. Both SIB1 (VQ16) and SIB2 (VQ23) contain a short VQ motif that is important for interaction with WRKY33. Transcripts associated with *WRKY33*, *VQ16* and *VQ23* homologues were enriched at 3 dai in the ILs (Fig. 5). Comparative transcriptomic analysis identified all 3 copies of *VQ16* were suppressed in the *Lm* infected Topas and Westar plants. Figure 5 presents a model describing the possible role of VQ proteins and WRKY genes in the induction and suppression of defense against *Lm*. Quantification of expression of *VQ16*, *WRKY33* and *PDF1.2* were conducted by Droplet Digital PCR (ddPCR) which confirmed the RNAseq expression data (Supp. Fig. 7).

While monitoring the receptor complex-associated *SOBIR1* homologues, it was noted that these were strongly expressed in T-*LepR1* and T-*Rlm2*, with expression peaking early in the time course (3 dai). It was previously reported that the *Arabidopsis thaliana* LRR-receptor-like kinase (LRR-RLK) suppressor of Bir-1(AtSOBIR1) interacts with LRR-RLPs resistance genes^57^. We have previously demonstrated that SOBIR1 binds with both Rlm2 and its allelic variant LepR3, which are membrane-bound receptor-like proteins (RLPs)^23,24^. This result suggests that SOBIR1 is also required for successful LepR1 defense response, and that *LepR1* may also encode an RLP. Conversely, very low expression of the *SOBIR1* homologues was observed during the *Rlm3*-induced defence response, which could indicate that Rlm3 may function independent of SOBIR1.

Our data showed a repression of cytokinin (CK) responsive genes during early infection (3 dai) of *Lm* in Topas and resistance lines while the induction of CK was observed in Westar (Supp. Fig. 4). CK level in *Bn* cotyledons increases upon *Lm* infection^58^. The role of CK has been previously reported in various pathosystems. Some pathogenic fungi such as *Magnaporthe oryzae*, *Cladosporium fulvum*, *Ustilago maydis* or *Claviceps purpurea* produce CK to compromise the host defense^59–61^. A search for the genes involved in the CK pathway identified isopentenyltransferase (IPT) and adenosine kinase (AK) in the genome of *Lm*^58^. Previous observation that the level of CK was elevated in the *Lm* infected tissues needs to be further explored to determine the origin of CK (pathogen or host) and its importance in defense against *Lm*.

Previous Quantitative trait locus (QTL) studies suggested a role for cysteine-rich protein kinase genes in quantitative resistance to blackleg disease in *Brassica napus*^62^. Association of CRK11 within a functional network of plant immunity related genes (based on DEG at 3 dai) further supports its importance in defence against *Lm* (Supp. Fig. 8). In the case of quantitative resistance to pathogens, additive effect of genes and the combined interaction of host genotype background and environment account for variation in phenotypes. However, immunity response triggered by single *R* genes is generally thought to be less variable. By taking advantage of *R* gene introgression lines described previously^13^ we show that, despite the involvement of the same pathogen effector and plant R proteins in triggering the initial immunity response, there are clear differences in the dynamics of defense related gene expression and this is influenced by the host genetic background. Robust immune response and arrest of the pathogen at the site of penetration is highly desirable when developing resistant cultivars. Furthering our understanding of how *R* genes interact with host genotype background will help ensure selection of the best germplasm for robust expression of resistance.

## Method and Materials

### Plant growth condition and pathogen inoculation

Plant material comprised canola introgression lines (ILs) containing (i) *LepR1*, *LepR2, Rlm2* and *Rlm3*, in Topas background (T-*LepR1*, T-*LepR2*, T-*Rlm2*, T-*Rlm3*), (ii) *LepR1* and *LepR2*, in Westar-N-o-1 background (W-*LepR1*, W-*LepR2*), and *Brassica napus* cv. Topas (Topas DH16516) and Westar N-o-1, without known resistance genes^13^. Plants were grown in the growth chamber at 20 °C, 16 h light, with the light intensity c. 450 μmol m^−2^ s^−1^ at the bench level, and 18 °C, 8 h dark. For fungal inoculation, a small wound was made in the center of each cotyledon lobe (four wounds per plant) of 7 day-old seedlings and 10 μL of 2 × 10^7^ spores/mL suspension was applied to each wound. In each of three experimental replications, half the one week-old plants from each IL, Topas and Westar (60 plants per line per condition) were inoculated with pycnidiospores of *Lm* isolate 00–100. The remaining seedlings were “mock” inoculated in the same manner with H_2_O.

### RNA isolation and RNA sequencing

RNA was prepared from three biological replicates of infected and mock-inoculated cotyledons. Twelve discs per sample were collected using a standard hole punch and pooled, with each disc centred on the initial wound site. Samples were snap frozen in liquid nitrogen for later extraction. For RNA isolation, samples were ground in liquid nitrogen, then extracted with TRIzol LS reagent (Invitrogen) and purified by applying to the Ambion mini RNA kit following the manufacturer’s instructions. RNA was DNAase treated, quantified by Qubit fluorometer (Invitrogen) and checked for quality by Agilent Bioanalyzer 2100 (Agilent Technologies). Only samples with RNA integrity numbers above 8.0 were used for sequencing. Sequence reads (100 bp paired-end) were generated with Illumina TruSeq- high output version 3 chemistry on a HiSeq 2500 (Illumina, Inc.) at NRC-Plant Biotechnology Institute (NRC*-*PBI), Saskatoon, Saskatchewan, Canada.

### Read mapping and analysis of RNA-Seq data

Transcriptomic analyses were carried out based on the methods described previously^4^. In total, 1.5 billion raw reads were analyzed for 168 samples in this study. Reads were trimmed, adaptor sequences were removed using Trimmomatic (http://www.usadellab.org/cms/?page=trimmomatic) and then mapped to the *B. napus* (Genome Resources – Genoscope)^63^ and *Lm*^19^ genomes using CLC Genomics Workbench (QIAGEN, Aarhus, Denmark). Transcript abundance was measured as log 2 RPKM (Reads Per Kilobase of exon per Million mapped reads). Genes with a false discovery rate (FDR-BH) less than 0.05 were considered differentially expressed (DEG) using the empirical analysis of DGE Tool, which implements edgeR analysis^64^ and DESeq2 package^65^. Principal component analysis (PCA) was performed to assess the variability among samples.

### Droplet Digital PCR (ddPCR) analysis

Quantification of expression of *WRKY33*, *PDF1.2* and *VQ16* were conducted by Droplet Digital PCR (ddPCR) according to the methods described previously^66^. RNA of *Lm* infected and mock inoculated Topas and Westar (from Group I), W-*LepR1* (from Group II), T-*LepR2* (from Group III) and T-*LepR1* (from Group VI) at 3, 6, 9 dai were adjusted to 1 μg of RNA, and cDNA from three biological replicates was synthesized using an iScript™ Advanced cDNA Synthesis Kit according to the manufacturer’s protocol (Bio‐RAD). All primers and probes (Supp. Table 2) were designed using “Quest tool” complimented by IDT (https://www.idtdna.com/PrimerQuest/Home/Index) and “BnActin” was used as reference. ddPCR was performed using a QX100 Droplet Digital PCR (ddPCR™) System – Bio-Rad. The Bio-Rad QuantaSoft™ Analysis Pro (QuantaSoft AP) software was used to calculate the ratio signal of assay/Actin. In order to get RNAseq and ddPCR result comparable, heat map was generated based on: Log2 (Assay/Actin) (mean of replicates): inoculated with *Lm*/not inoculated control and Log2 RPKM (mean of replicates): inoculated with *Lm*/not inoculated control (Supp. Fig. 7).

### Functional classification based on Blast2Go and KEGG

Annotation of DEG was performed by Blast2Go-pro^67^ and then KEGG (Kyoto Encyclopedia of Genes and Genomes) enrichment analysis was done using KEGG function of Blast2Go-pro software to assign predicted pathways for DEG. HORMONOMETER^40^ was used to evaluate the transcriptome response through the perspective of similar events that happened upon hormonal activation in Arabidopsis.

### Functional network analysis

The putative *A. thaliana* orthologs of *Bn*-DEGs were identified and then considered for a functional network analysis. An integrated pathway and interaction data based on co-expressed, co-localized genes and gene ontology information was thus identified using GeneMANIA, which was implemented through Cytoscape. Transcriptional landscape was also generated using SeqEnrich^41^.

### Confocal microscopy: observation of *Lm* hyphae

To visualize *Lm* hyphae, the area 1 cm around the wound that was made in the center of each cotyledon of 7 day-old seedlings of ILs and susceptible lines was excised at 6 dai. The samples were soaked in a staining solution containing WGA-AlexaFluor488 (Invitrogen) at room temperature for 10 min. Observations with a TCS-SP5 confocal laser-scanning microscope were done at objectives of 20x. WGA–Alexa Fluor 488 was detected with a 488 nm excitation and 500–540 nm emission wavelength. Raw images were first deconvolved using AutoQuant X3 software (10 iterations) and then were imported into Imaris 7.4.1 to remove background noise.

## Supplementary information

Supplementary Figure1-8

Supplementary Table 1

Supplementary table 2
