# Supplementary material for: Dissecting R gene and host genetic background effect on the Brassica napus defense response to Leptosphaeria maculans
Source: Sci Rep. 2019 May 6;9:6947. doi: 10.1038/s41598-019-43419-9 (PMC6502879; doi:10.1038/s41598-019-43419-9)
Supplement: Supplementary file 1 — Supplementary Figure1-8 [file 41598_2019_43419_MOESM1_ESM.pdf]

Dissecting *R* gene and host genetic background effect on the *Brassica napus* defense response to *Leptosphaeria maculans*

Parham Haddadi[1], Nicholas J. Larkan[2], M. Hossein Borhan[1]

[1]Agriculture and Agri-Food Canada, Saskatoon Research and Development Centre, 107 Science Place, Saskatoon, SK Canada, S7N 0X2

[2]Armatus Genetics Inc., Saskatoon, SK, Canada, S7J 4M2

Corresponding author: hossein.borhan@agr.gc.ca

Supplementary Figures (legends)

Supp. Figure 1: Heatmaps present the difference in expression of *Brassica napus* genes with homology to (A) *SOBIR1* (B) *CRK 4, 5* and *20* as HR inducer (D) *CRK 2, 10, 11* which are involved in chitin immunity defense response in *Leptosphaeria maculans* inoculated compared with mock-treated cotyledons of Topas, Westar and ILs at 3, 6 and 9 dai. The colors correspond to log 2 RPKM (Infected – Uninfected) ranging from red (high) to green (low). Euclidean distance for the distances measure and complete linkage for clusters linkage criteria were selected.

Supp. Figure 2: Hormone-related transcript correlation associated with common up- regulated genes for all Introgression Lines (ILs) at 3, 6 and 9 dai. A positive correlation between the query (*Brassica. napus* differentially expressed genes) and the *Arabidopsis thaliana* genes expressed when treated with various plant hormones is denoted in red, whereas a negative correlation is represented by blue. Common genes are also shown for each comparison.

Supp. Figure 3: Heatmaps depict the difference in expression of *Brassica napus* genes with (A) *NIMIN 1,2* (B) genes with homology to known marker genes related to SA (*WRKY 70*, *ICS1*, *PR1*) and (C) chitinase (*CHI*) in *Leptosphaeria maculans i*noculated compared to mock-treated cotyledons of Topas , Westar and ILs at 3, 6 and 9 dai. The colors correspond to log 2 RPKM (Infected – Uninfected) ranging from red (high) to green (low). Euclidean distance for the distances measure and complete linkage for clusters linkage criteria were selected.

Supp. Figure 4: Hormone-related transcript correlation associated with *LepR1*, *LepR2*, *Rlm2* and *Rlm3*. A positive correlation between the query (*Brassica napus* differentially expressed genes) and the *Arabidopsis thaliana* genes expressed when treated with various plant hormones is denoted in red, whereas a negative correlation is represented by blue. Common genes are also shown for each comparison. Data revealed the repression of Cytokinin in all lines so it could be considered as a negative regulator. Distinct cluster was also observed for *LepR1* and *RLM2* in Topas background at the earlier stages.

Supp. Figure 5: Transcriptional landscape at early stage of *Leptosphaeria maculans* infection in (A) Topas and (B) Westar.

Supp. Figure 6: (A) Pattern-triggered immunity (PTI) and effector-triggered immunity (ETI) to counter *Leptosphaeria maculans* (*Lm*) invasion in Topas, Westar and Introgression Lines (ILs). Intensity scale: red represents more differential expressed genes corresponding to PTI and ETI in response to *Lm.* (B) Heatmaps present the difference in expression of *Brassica napus* genes with homology to *NDR1* in *Lm*-inoculated compared to mock-treated cotyledons of Topas, Westar resistance lines at 3, 6 and 9 dai.

Supp. Figure 7: Quantification of expression of *WRKY33*, *PDF1.2* and *VQ16* using Droplet Digital PCR (ddPCR).

Supp. Figure 8: CRK11 as main defense component in canola-blackleg interaction. CRK11 come up when a functional network associated with immune system process in resistance lines generated through earlier up-regulated genes (DEGs-at 3 dai).

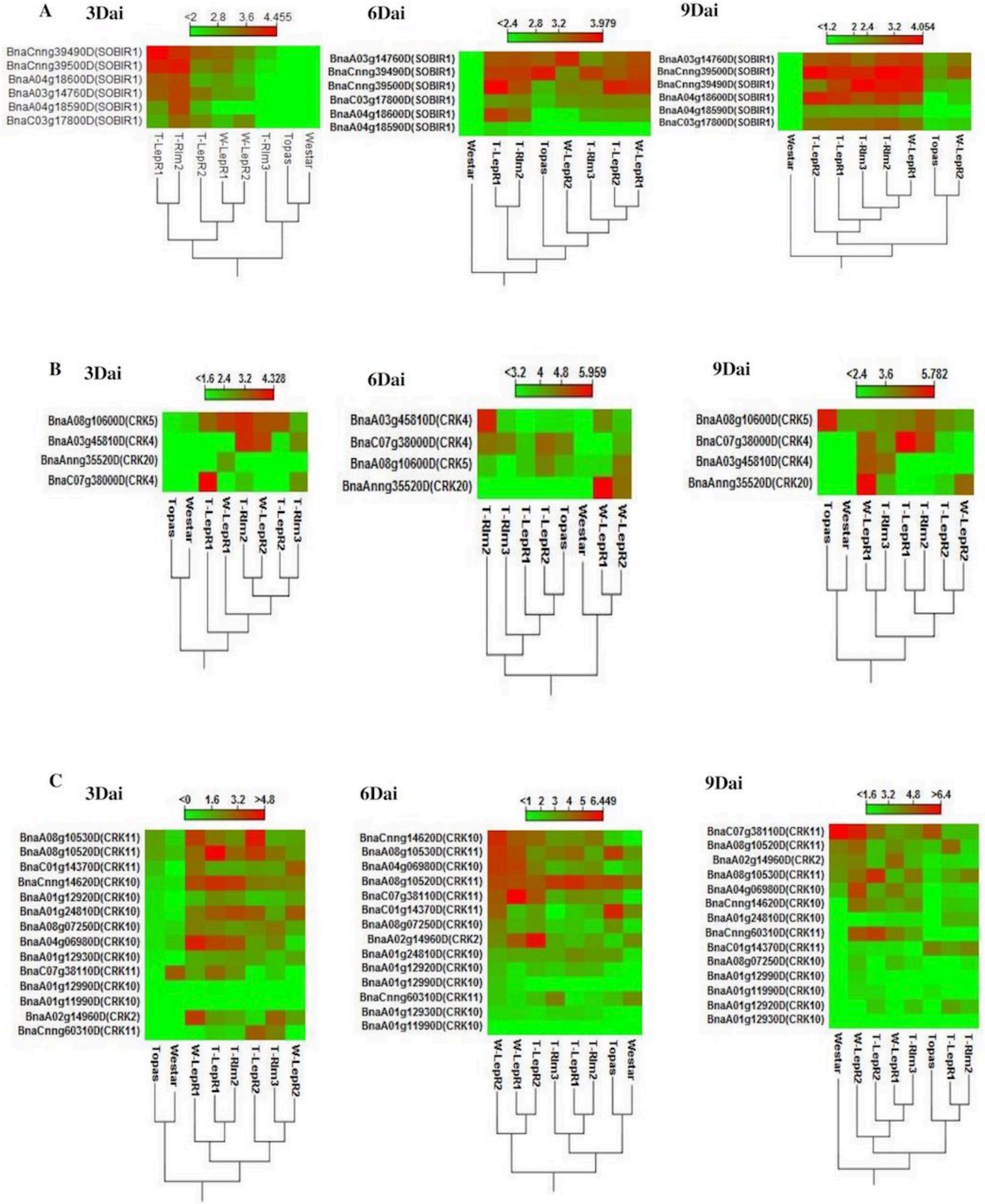

Supp. Figure 1

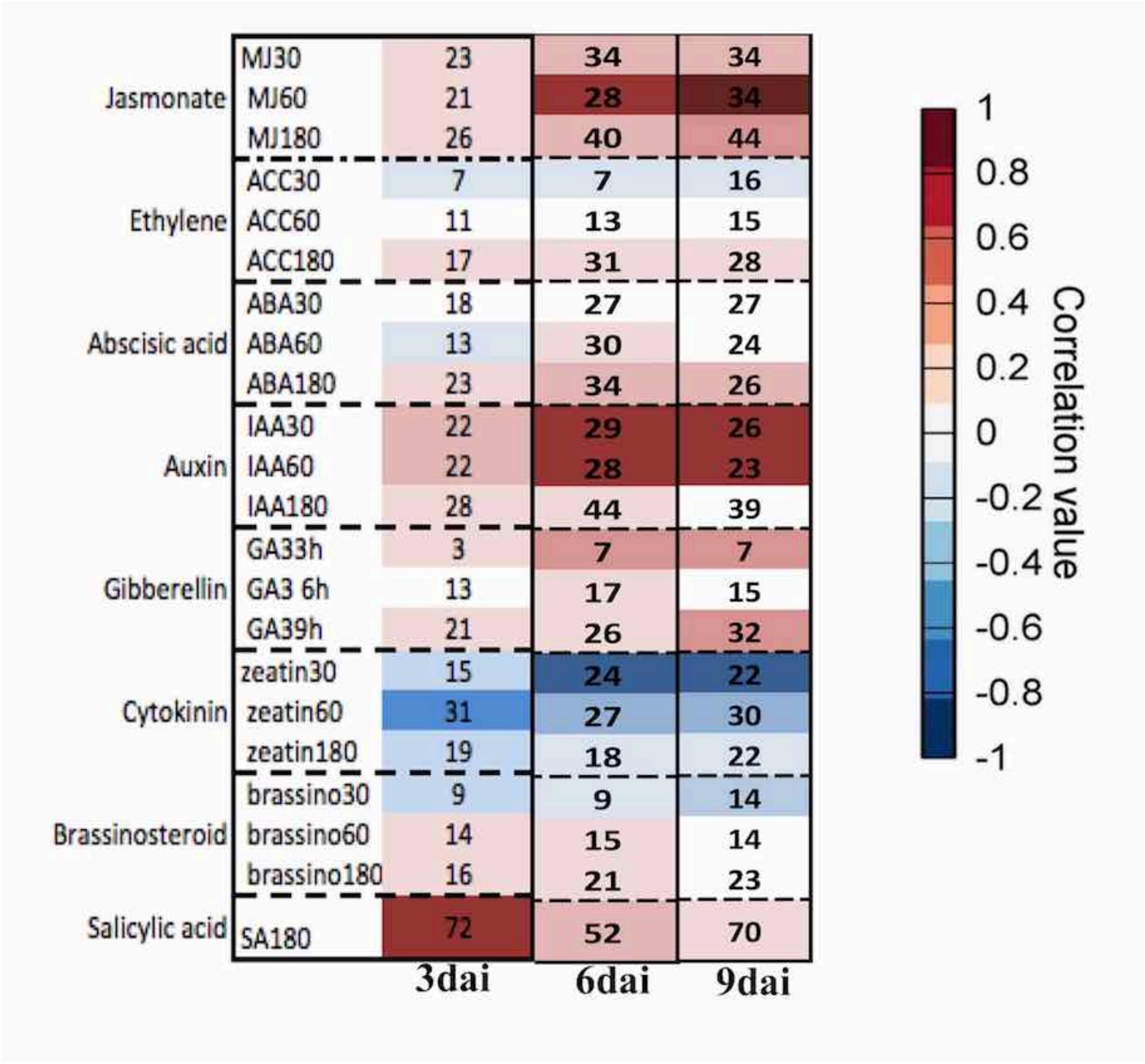

Supp. Figure 2

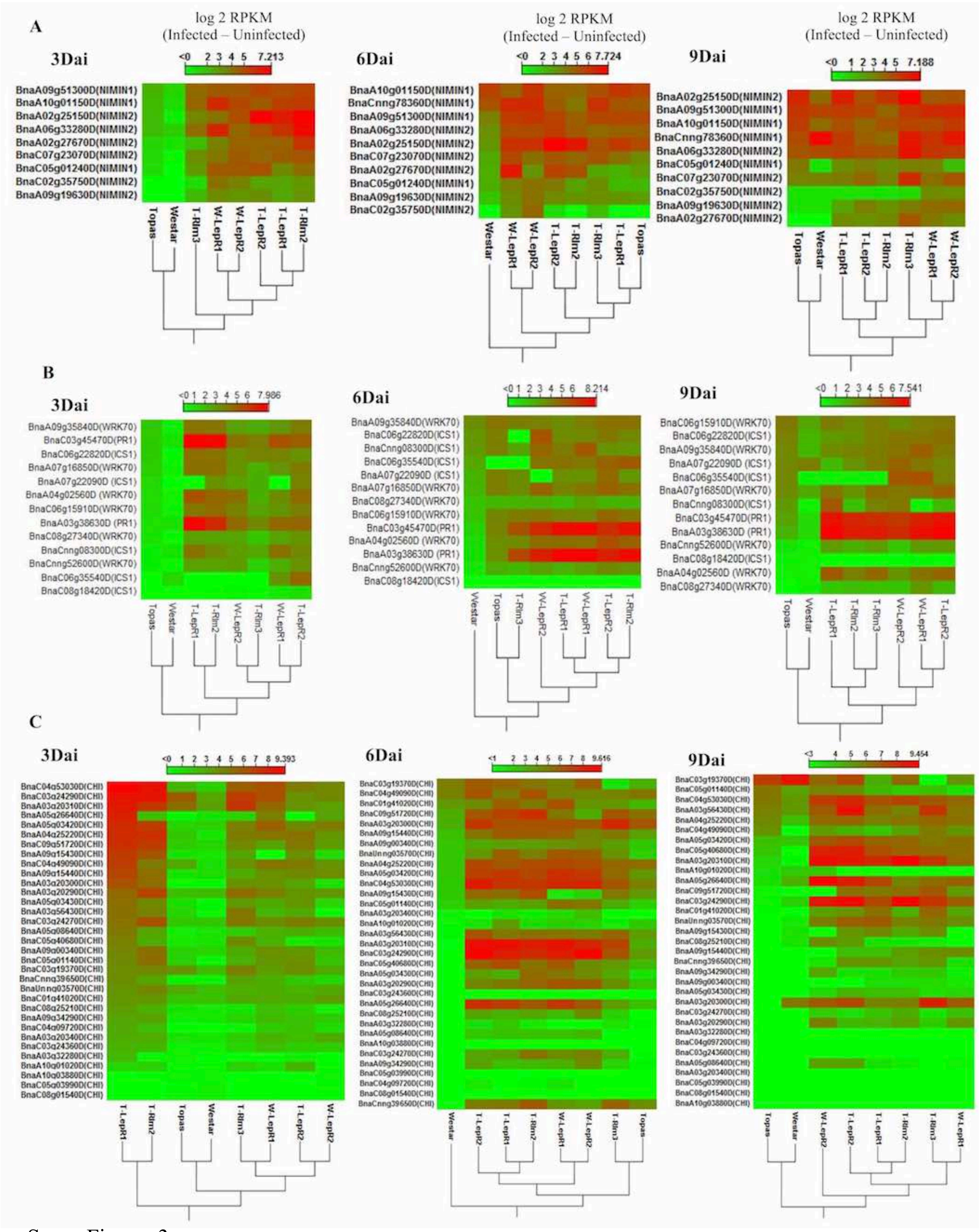

Supp. Figu re 3

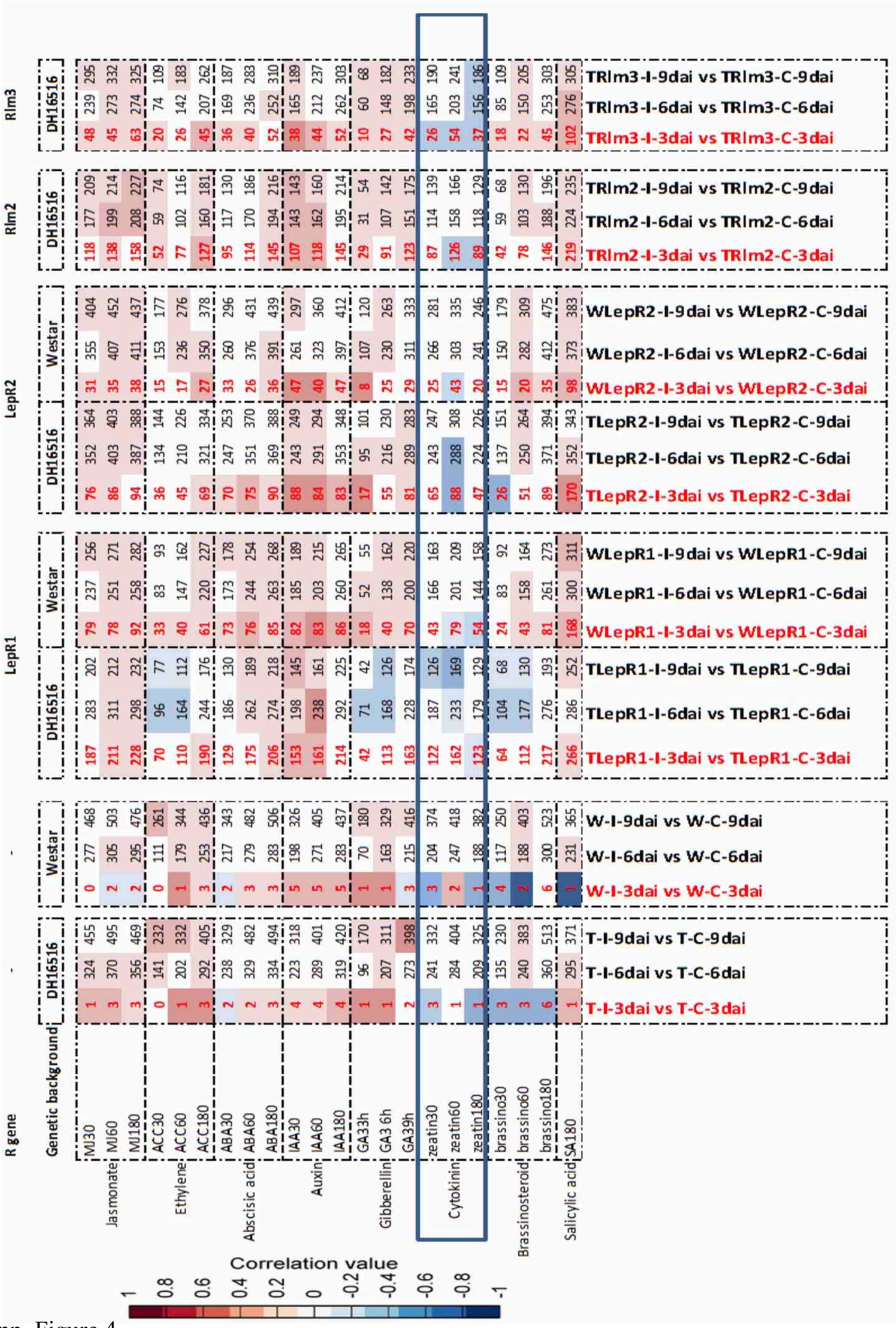

Supp. Figure 4

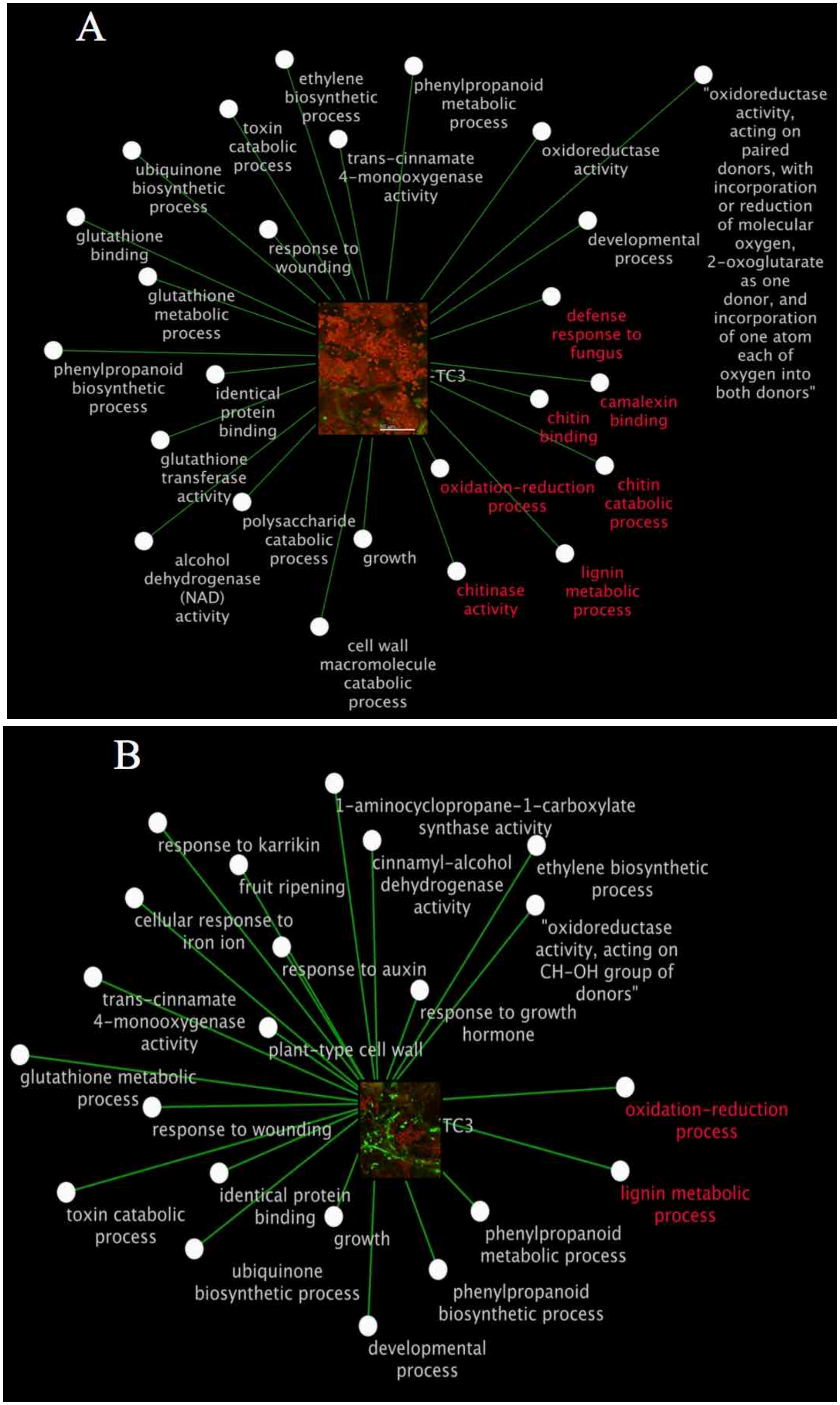

Supp. Figure 5

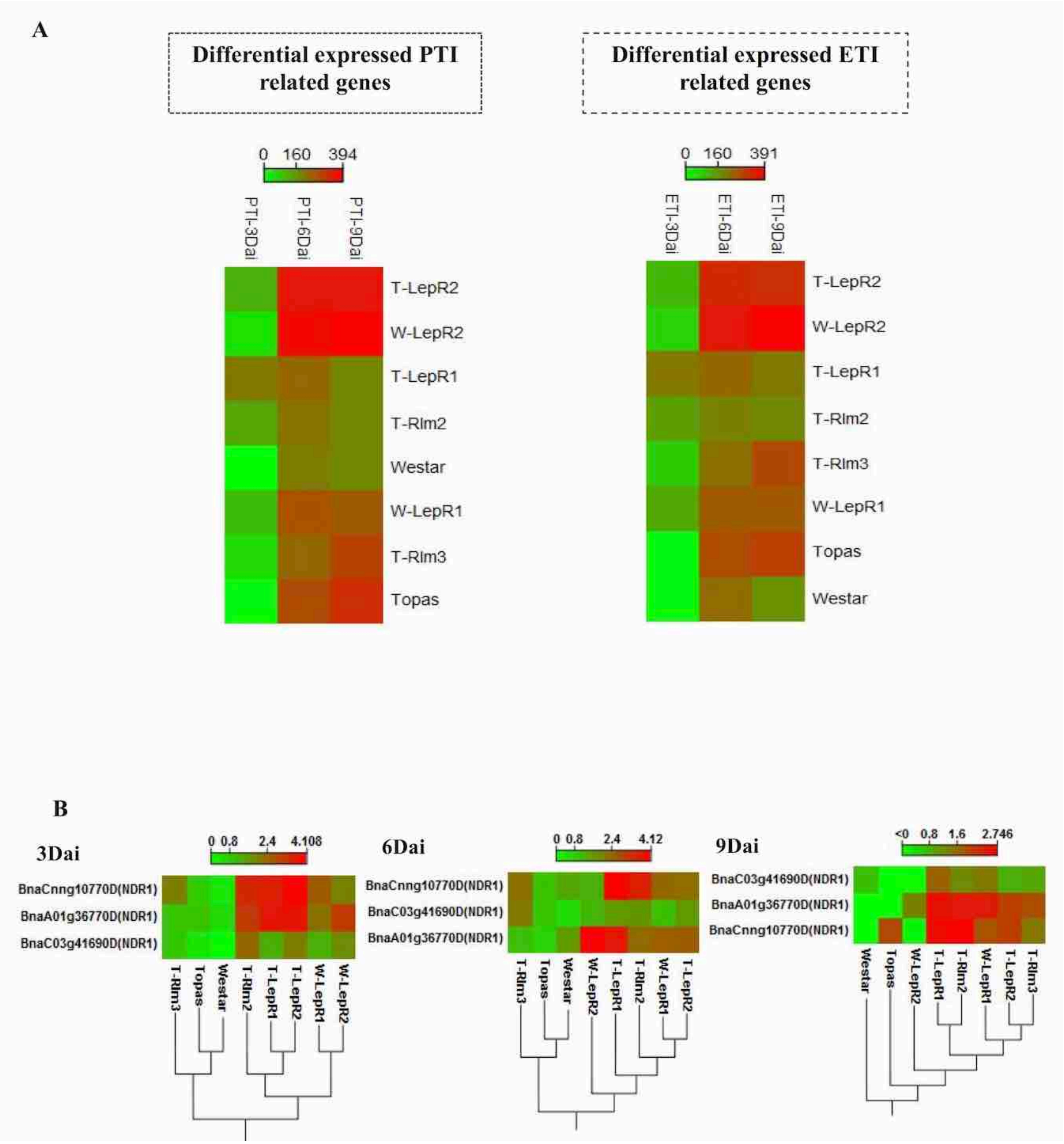

Supp. Figure 6

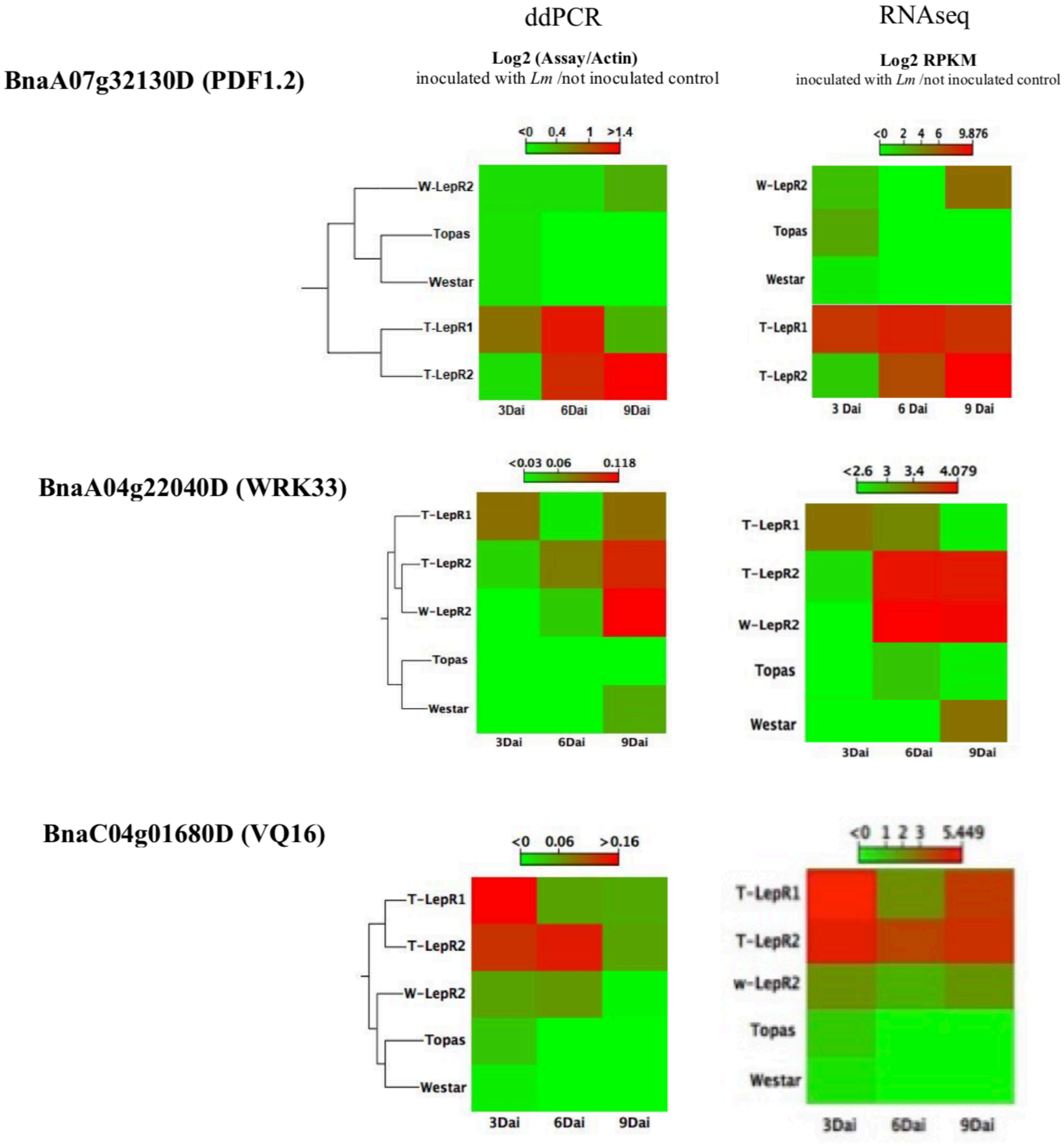

Supp. Figure 7

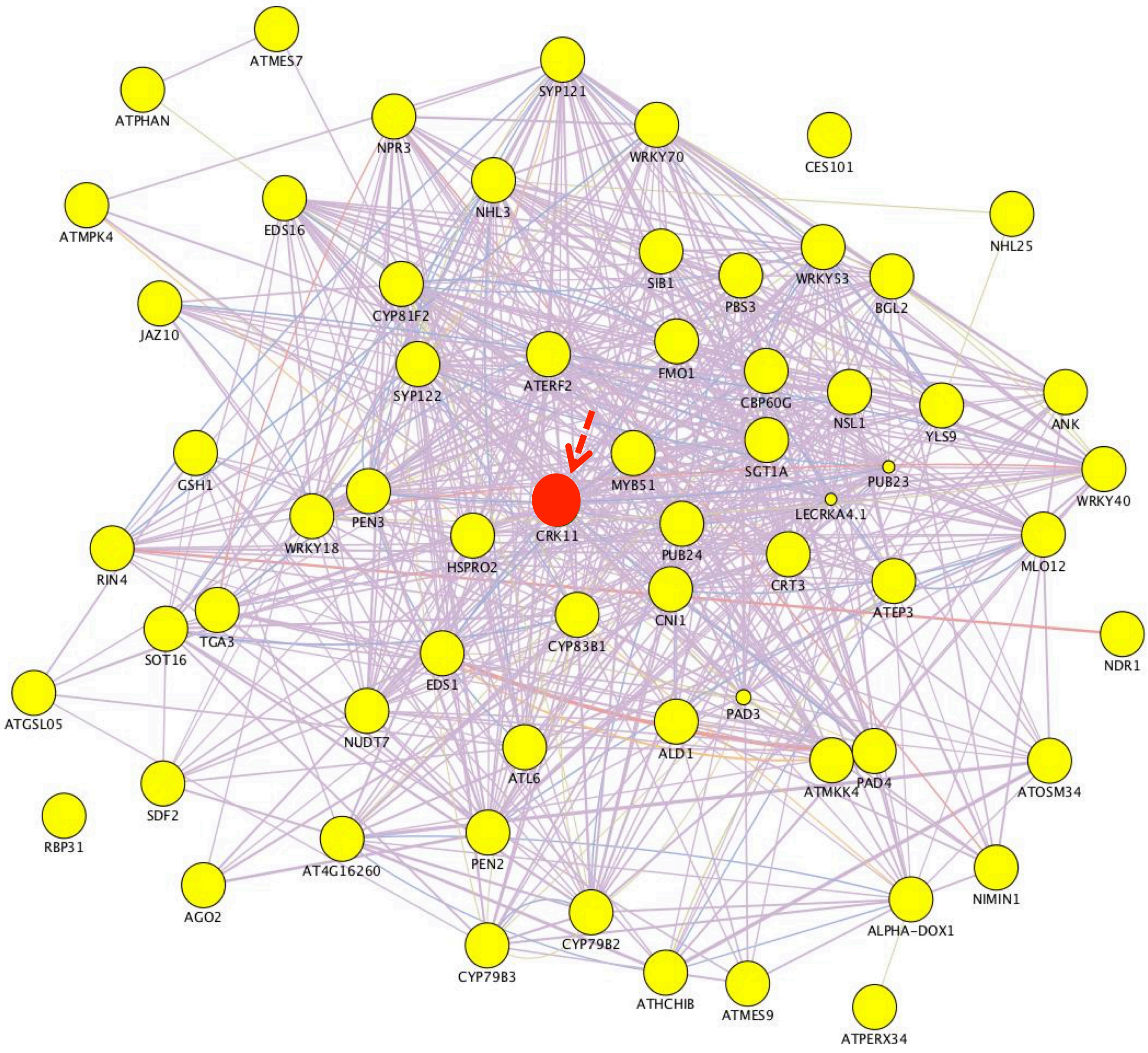

Supp. Figure 8
